# Participation as a means-implications for intervention reasoning

**DOI:** 10.3389/fresc.2024.1399818

**Published:** 2024-06-27

**Authors:** Mats Granlund, Christine Imms

**Affiliations:** ^1^ School of Health and Welfare, Jönköping University, Jönköping, Sweden; ^2^ Medicine, Dentistry and Health Sciences, The University of Melbourne, Melbourne, VIC, Australia

**Keywords:** participation, inclusion, intervention, rehabilitation, children

## Abstract

**Introduction:**

The increased focus among researchers and professionals on participation as an explicit intervention outcome has prompted a paradigm shift in both thought and practice. However, much research centers on altering participation outcomes in specific life situations and stages. This discussion paper considers “participation as a means” in pediatric rehabilitation and special education interventions, emphasizing its role in achieving lasting outcomes.

**Method:**

This paper uses a Venn diagram approach to consider relations between three core concepts—participation, intervention, and outcomes—and their intersection. The paper's central theme revolves around the intersection of these concepts, wherein participation serves as a means to achieve enduring participation outcomes within the realms of rehabilitation and special education. The discussion is supported by contemporary empirical work and from literature identified in two recent scoping reviews focusing on the intervention process.

**Results:**

Achieving enduring participation outcomes through participation in the intervention process necessitates creating a learning experience, with children and families actively participating in every step: identifying participation issues, seeking explanations, prioritizing intervention goals, selecting methods, implementing interventions, and evaluating the process and outcomes.

**Discussion:**

This structured approach supports professionals and researchers to foster the skills and capacity required for lasting participation outcomes for children with impairments.

## 1 Introduction

Over the past two decades, a growing awareness of participation interventions has emerged among researchers and professionals. Conventional emphasis on skills training in pediatric rehabilitation and special education doesn't consistently result in increased participation in everyday activities for children with impairments (1). This recognition sparked a “paradigm shift” (2) where participation, defined as involvement in a life situation (3), is now the explicit desired outcome of interventions, rather than an implicit goal within skills training. This paradigm shift involves not only participation as an outcome but also participatory approaches to the intervention process itself (2). While participation outcomes post-intervention are beneficial for the individual (4), they also raise another question: Does increased participation in a specific activity following an intervention automatically lead to enhanced participation in other activities? The evaluation of whether one or more interventions, with participation in a specified activity as the desired outcome, influences the overall level of participation across different contexts is infrequent. Interventions focused on involving the person with disability in the whole intervention process provide some initial support (5). It's likely that the cumulative effect of a series of interventions on overall participation necessitates viewing participation not solely as an outcome but also as a means.

Participation is inherently context-dependent, and increasing an individual's participation in a specific activity can be achieved by adapting the environment to suit their needs or by providing support through skill development or assistive technology. To enable individuals to construct their preferred activities and participate autonomously, we must consider motivation, skills, and knowledge required for facilitating participation across multiple contexts. Once necessary skills and knowledge for autonomous problem-solving regarding participation are identified, we can explore the essential elements for teaching and cultivating these skills, e.g., learning through doing or experiencing.

Experiences from person-focused and family-centered interventions—that use participatory strategies for collaborative problem-solving as their basis—suggest that active involvement in the problem-solving process is an effective means for skill development (6, 7). Participation in intervention processes serves as a mechanism enhancing problem-solving skills in most interventions. Therefore, it is important to understand how the features of the different steps in the intervention process can enhance the learning aspects of the process.

This article explores participation as a means within pediatric rehabilitation and special education interventions. We suggest that: by conceptualizing participation as an enduring outcome of interventions, participation in the intervention process can be viewed as “a means to an end” within a series of participation interventions.

The paper's aim is to introduce and discuss a structured approach to implementing the intervention process that utilizes participation in rehabilitation interventions to foster the necessary skills for lasting participation outcomes in children with impairments. We propose that future research should address the question of whether using participation as a means in rehabilitation interventions can support development of enduring participation outcomes for children growing up with disability.

## 2 Method

**Figure 1:**
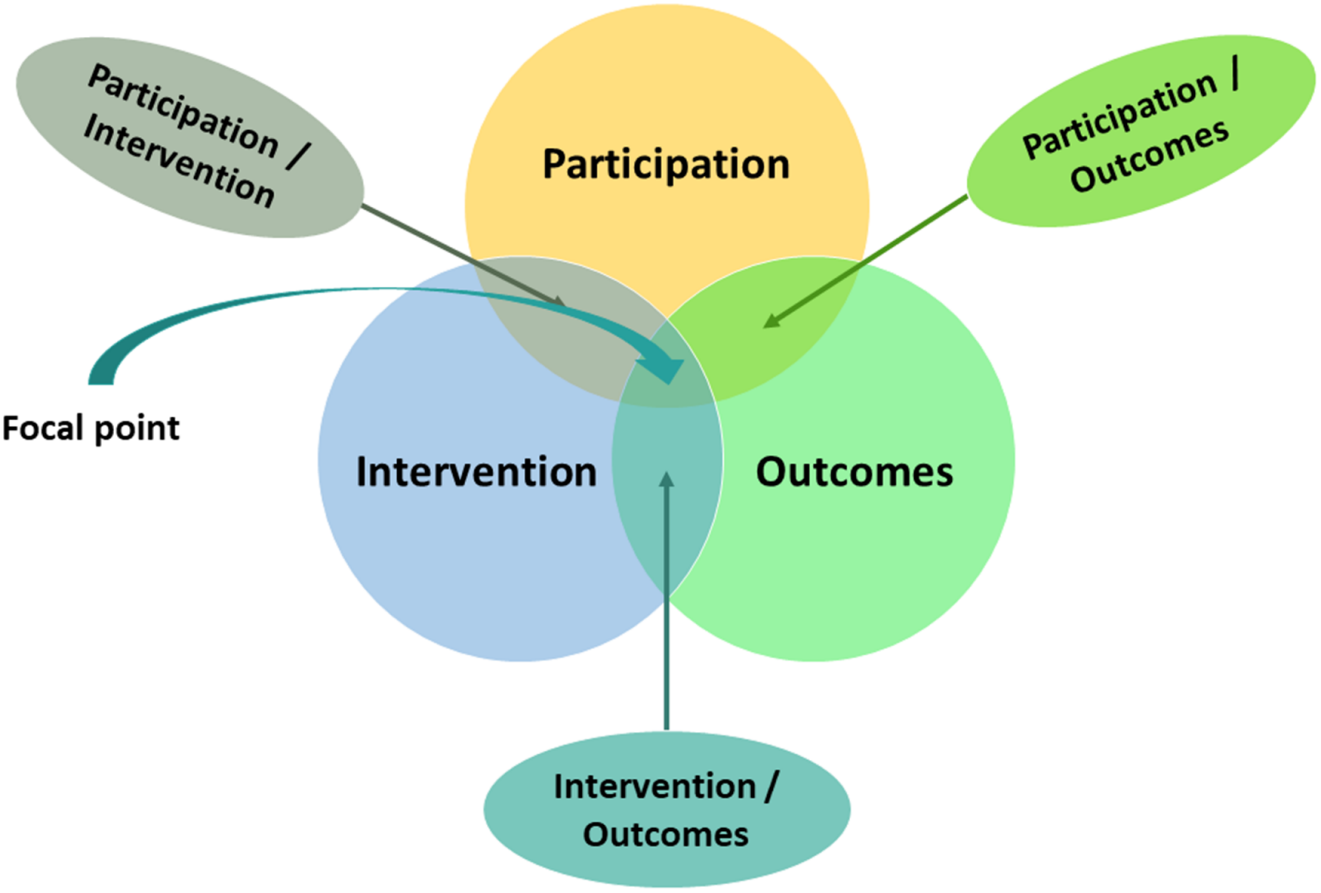
Overview of the structure of the paper, showing three central concepts, participation, intervention and outcomes, and how they intersect. The central “focal point” is the intersection among all three concepts, where participation as means—within rehabilitation—is used to drive enduring participation outcomes.

In this discussion paper, the content has been organized using a Venn diagram approach, as illustrated in Figure 1. Venn diagrams are used to explore potential overlaps and relationships between and among concepts (8). They provide a structure for organizing information and thinking. In this paper we chose the Venn diagram to support describing our proposed framework for how to embed “participation as means to participation outcomes” in paediatric rehabilitation. Initially we define the three concepts of interest—participation, intervention, and outcomes—each of these concepts has its own large body of evidence. We are interested in how the concepts overlap. Therefore, we next describe three areas of intersection or overlap between the pairs of concepts: participation/intervention, participation/outcome, and intervention/outcome, informed by known literature. Finally, we present and discuss a structured rehabilitation framework focusing on the central intersection of participation-intervention-outcome, serving as a proposed method for achieving lasting participation outcomes for children and their families. The arguments and considerations for the participation-focused rehabilitation framework are supported by contemporary empirical research, including evidence from a recently published scoping review on professionals’ use of strategies to enhance children's engagement in the intervention process (9) and a scoping review (in preparation) concerning interventions that aim to enhance child and care provider involvement in the intervention process in pediatric rehabilitation. The evidence cited is not the product of one systematic review of the literature, but rather is used as exemplars, to support or pose questions about the concepts and framework proposed.

## 3 Part 1. Defining the concepts and conceptual overlaps

### 3.1 Participation

Under the United Nations Convention on the Rights of Persons with Disability (10), all people have a right to full and effective participation and inclusion in society. While the UNCRPD does not explicitly define participation, nor inclusion, the principles of the Convention (as articulated in Article 3) describe the conditions for good participatory practices. These include respecting people's inherent dignity, enabling autonomy and ensuring there is freedom to make one's own choices, equality of opportunity, ensuring accessibility, and respect for people's evolving capacities (10). The UN Convention on the Rights of the Child (11), also includes the right to participation and inclusion. The UNCRC has underpinned participatory models for children like that proposed by Lundy (12). Lundy's participatory framework focuses on the requisite elements for ensuring children's rights to have influence over decisions affecting them, including a safe space and opportunity, voice, audience and influence. One area where children's rights to be listened to is the intervention process in pediatric rehabilitation.

The Declaration of Human Rights in 1948, along with the subsequent Conventions on Rights, provide the foundations for all participatory practices, including paediatric rehabilitation. In 2001, the World Health Organisation first included the word “participation” in its frameworks for health, when the International Classification of Functioning, Disability and Health (ICF) was published (13). In the ICF, participation is defined as involvement in a life situation, inherently contextualized and influenced by personal, environmental, and contextual factors, each of which may become the focus of intervention methods. Life situations include the day-to-day activities that make up a life—including, but not limited to, decision-making. Based on ICF, the Family of Participation Related constructs (fPRC), was developed to support research and clinical practices by providing a framework for participation reasoning and empirical testing (14). Central to the concept of participation in fPRC are two dimensions: attendance and involvement in life situations (14). Attendance, whether in the physical or virtual realm, is a prerequisite for involvement. When attending, the degree of involvement may vary, encompassing various subdimensions of involvement like engagement in the activity, a sense of belonging, and the perceived importance of the activity (14).

The two dimensions of “participation” in the fPRC extend to individuals within their living environment (family, school, work, leisure, society). They also apply to interactions between individuals with impairments and those providing support in settings like child/family/professional encounters, as well as evaluating universal conditions for participation in service systems and society. The interaction between children, care providers and professionals is the main focus for this discussion paper. Within the fPRC framework (14), the two dimensions of participation—attendance and involvement—are, at the level of the individual, linked to intrinsic and extrinsic factors presumed impacting participation. Intrinsic factors encompass the individual's activity competence (e.g., knowledge and skills), their sense of self (e.g., self-efficacy or self-determination), and preferences (e.g., interests). Extrinsic factors encompass the context actively constructed by the person in interaction with the environment during the activity and the environment existing independent to the person. At the level of interactions between an individual, care providers and the service systems, intrinsic factors in the child and care providers still relate to activity competence, skills, sense of self and preferences of the child and care providers. Extrinsic factors concern the interaction with professionals in the context, the context itself (e.g., pediatric rehabilitation) and the skills, values and attitudes of professionals. These interactions among children, family members and professionals primarily take place in the process of provision of services or interventions.

#### 3.1.1 Interventions

Intervention, as per (15), can be defined as intentional actions taken to achieve a desired outcome, whether singular or part of a series of interventions. The concept of an “intervention process” evolved in recent years, expanding beyond the sole focus on evaluating the effects of specific intervention methods. Presently, the intervention process is recognized as encompassing six generic steps, ranging from problem identification to outcome evaluation (see Table 1). These steps are applicable to all types of interventions, including those in public health, epidemiology, or targeting specific individuals or groups (16). Intervention commences as soon as significant challenges requiring intervention are identified or expressed by individuals or professionals and concludes with the assessment of the intervention's effects on the outcome.

**Table 1:** The six steps of the intervention process.

Step	Process
i	Assessment/problem definition
ii	Hypothetical explanations (causes) to the problem
iii	Prioritizing what to work with—goal setting
iv	Designing/choosing the intervention method
v	Implementation
vi	Evaluation of implementation and outcomes

**Figure 2:**
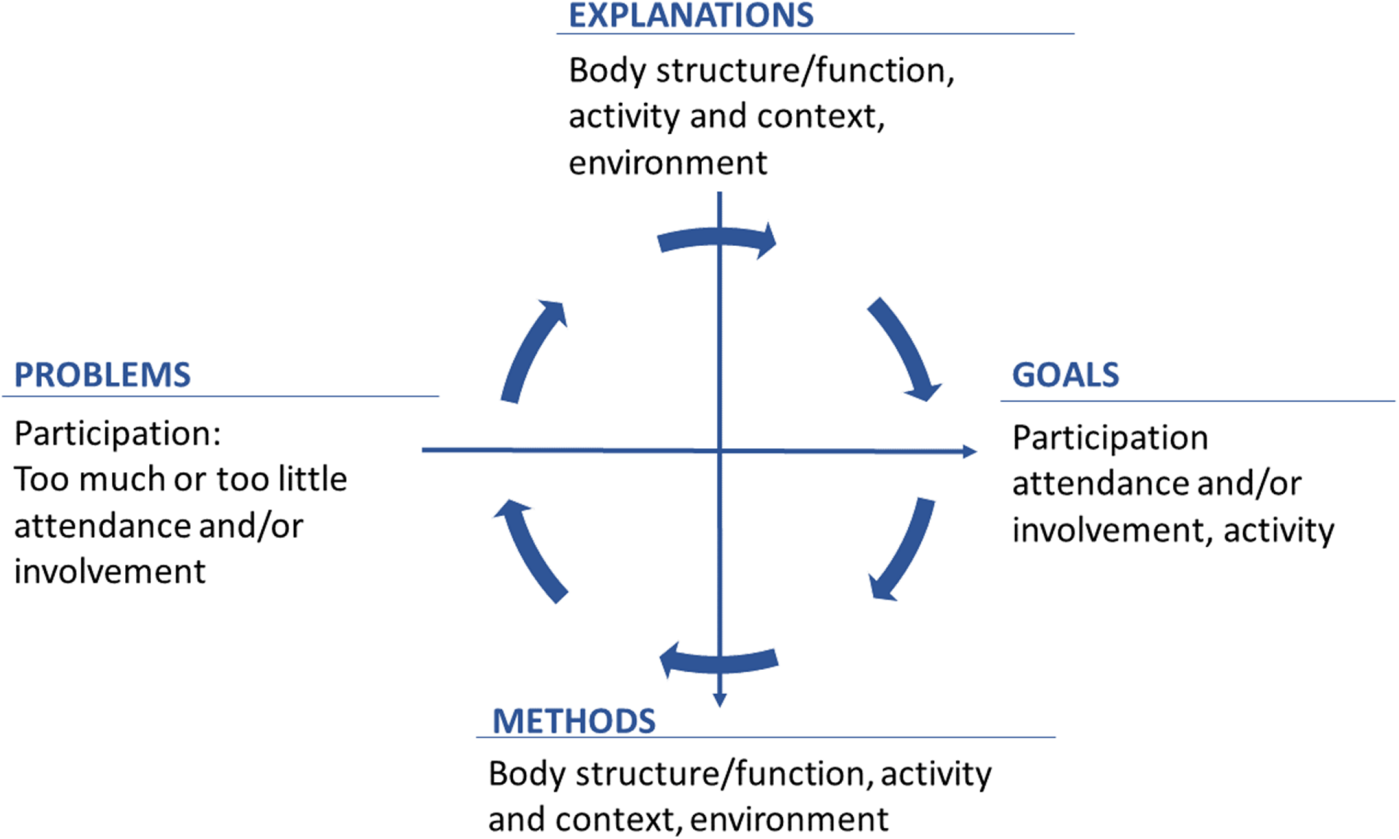
Planning, implementing and evaluating one intervention cycle.

Figure 2 illustrates an intervention cycle. For instance, the issue requiring intervention could involve low attendance or limited involvement in a crucial activity. Potential explanations or causes for this problem can be identified within the factors thought to influence participation. The goal is to reach a state where the problem no longer exists, signifying the desired level of participation. The method involves utilizing the hypothesized explanations formulating intervention strategies and putting them into practice. Subsequently, the evaluation seeks to determine if the method was successfully implemented and if the goal was achieved. Children receiving pediatric rehabilitation are exposed to recurring intervention cycles, of various kinds, for long time periods, sometimes throughout their whole life. Pediatric rehabilitation can be considered a complex intervention (17) containing many interventions (e.g., those delivered by different professionals) that might occur simultaneously. For each intervention, however, there is an intervention cycle.

#### 3.1.2 Outcomes

Outcomes, also known as effects or the objectives of interventions, can be defined as changes in behavior, attitudes, relationships, or the environment within a specified timeframe due to particular causes or planned interventions (15). Most effects result from multiple causes, some are essential (always present), others are not (18). One contributing factor to change is interventions, yet interventions occur within broader settings that also influence the outcome.

Ideally, the objective of an intervention aligns with the desired outcome. However, often the intervention's goal is implicitly related to the long-term desired outcome. Schlosser and Braun (19) present three potential ways to link an intervention goal with a long-term desired outcome: (i) Intermediate goals: Outcomes facilitating subsequent interventions aimed at achieving long-term outcome. (ii) Instrumental goals: Interventions expected to lead to long-term outcomes different from those initially targeted in the intervention. (iii) Ultimate goals: Enduring, intrinsically significant outcomes in and of themselves (19).

These categories of goals can be employed to categorize objectives for participation interventions, with only ultimate goals explicitly centering on participation outcomes (effects). Nevertheless, effects always occur within a timeframe, allowing them to be positioned on a spectrum from momentary effects to persistent effects. Simeonsson (20) outlined these terms as follows: (i) Transient effects—outcomes specific to a particular time, context, and task. (ii) Enduring effects—outcomes that persist over time, encompassing various contexts and tasks.

### 3.2 Exploring the interactions between concepts

The intersections between these concepts primarily revolve around the interactions between various aspects, such as how participation factors interact with outcomes.

#### 3.2.1 Participation-outcomes interactions

When examining the interaction between participation and outcomes, it's noteworthy that many participation goals in interventions often take the form of intermediate or implicit instrumental objectives. Intermediate goals intend to facilitate subsequent interventions with participation as the desired outcome. For example, training children to use an eye gaze system to later enhance their use of graphic symbols for communication (21). Implicit goal setting occurs when we establish goals for skills training with the implicit assumption that the child will engage more in participation when employing the trained skills.

Explicit participation goals, evident in current literature, predominantly concentrate on increasing frequency, duration, or variety of activities attended. There remains a need for a deeper understanding of interventions aimed at elevating involvement during attendance (22). Set goals tend to be transient, confined to specific timeframes, contexts, and tasks; for instance, increasing the frequency of attending after-school football/soccer training (23, 24). Is it feasible to establish enduring individual participation goals for interventions? Or, is the enduring goal better directed towards instilling the necessary skills and self-identity to engage in a diverse array of activities across various environments and life roles? The aim is to ensure individuals possess the personal competencies and environmental opportunities required for participation. Table 2, drawing from Schlosser and Braun (19) and Simeonsson (20), illustrates concepts in the form of an outcomes matrix. These ideas closely align with the definition of health proposed by Huber et al. (25), which posits that “health is the ability to adapt and self-manage”, and with Bickenbach et al. (26) that suggest that besides mortality and morbidity one aspect of health is functioning.

**Table 2:** An outcomes matrix for participation effects.

Effects	Goals		
	Intermediate: facilitate further intervention	Instrumental: expected to lead to other outcomes than those specified	Ultimate: long lasting and important in and by themselves
Transient: specific in time, context and task	e.g., skills in operating synthetic speech devise to facilitate communication intervention aimed at increasing social interactions.	e.g., skills in walking and fine motor grasp to “automatically” increase participation in outdoor play activities; or, enhancing attending and/or involvement in outdoor play activity to “automatically” increase skills.	e.g., enhancing attending and/or involvement in outdoor play activities. Involvement can be engagement or sense of belonging in outdoor play activities.
Enduring: broad, cover many contexts and tasks	e.g., skills in identifying environmental facilitators and barriers for participation in natural environments.	e.g., skills in in self-advocacy as applied in natural settings or in planning participation interventions in collaboration with professionals.	A person that participates in a diversity of activities based on interests, personal obligations and societal demands from a life-long perspective.

#### 3.2.2 Participation-intervention interaction

**Figure 3:**
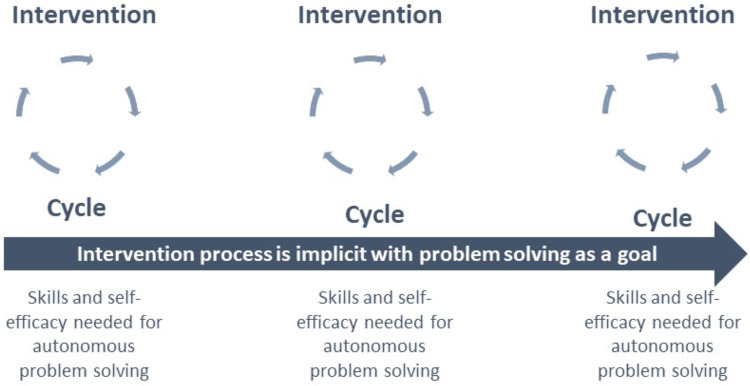
Participation intervention as a process over time. The effect of using “participation as means” within intervention circles is shown as a process over time to build autonomy in solving participation problems.

To enable children to attain autonomy in their participation across diverse domains of activities and life roles, requires (re)habilitation and education to focus on empowering them to independently construct participation. As per Knapp (27), “Interventions seldom consist of only one intervention or a specific series of interventions. A more accurate description may be a set of potential interventions influenced by factors that shape the interaction between a client/family and these interventions.” This description underscores the significance of both individual interventions and the entirety of potential interventions. One method for supporting children to develop into independent problem solvers, is to repeatedly involve them in all phases of the intervention process in as many of the ongoing interventions as possible. Consequently, as depicted in Figure 3, we propose that it will only be by examining participation aspects at each step within the intervention process, at various time points and longitudinally, that we will be able to accumulate knowledge capable of enhancing the comprehensive impacts of participation interventions. The emphasis lies in viewing participation in the intervention process as a means to equip individuals with the expertise to construct their own participation.

Participation in one or multiple activities can serve to enhance a child's skill development (activity) or even bodily functions. Anaby et al.'s study (2020) provides evidence of using participation as an instrumental goal to achieve objectives related to skill development. The rationale for this effect is that natural acquisition of new knowledge and skills necessitates spending sufficient time engaged in motivating activities fosters high levels of engagement and a sense of belonging within that context.

Moreover, participation can also function as a method for honing skills and cultivating a robust sense of self to enhance autonomy in future participation scenarios. This has been illustrated by Kramer et al. (28), where individuals with impairments build skills in assessing environmental prerequisites and facilitators for participation, in addition to acquiring self-advocacy skills that enable them to adapt activities to optimize participation. The rationale behind this approach is to pursue and tailor activities in line with personal aspirations, individuals must possess the ability to self-regulate their behavior in response to environmental conditions. They also need to adapt their surroundings to suit their needs, often in collaboration with others.

#### 3.2.3 Intervention-outcome interaction

Ideally, the intervention's goal should align with the desired outcome. Participation, being a contextualized outcome, underscores the importance of involving the individuals targeted by the intervention in specifying desired objectives. When addressing participation outcomes of interventions, it's crucial to consider the perceptions of both child and care providers regarding participation (29), as their perspectives may not completely coincide. Furthermore, discussions with children and care providers should encompass potential outcomes beyond the intended ones.

A common issue leading to limited effectiveness in participation and disease prevention interventions is low adherence to planned interventions (30). Frequently, the problem lies in the delivery of the intervention itself. For instance, the intervention may be overly time-consuming, or the goals may not be self-selected, thereby lacking motivation. Regarding the intervention process as an intervention, the question arises: what should individuals learn or change because of their involvement in the intervention process, particularly concerning participation outcomes? This question assumes particular significance for individuals, such as those with impairments, who are subjected to long-term or lifelong interventions (25).

#### 3.2.4 Participation-intervention-outcome—the focal point

This paper centers on the intersection of three key concepts: participation, intervention, and outcome. Participation is inherently contextual, meaning its outcome in any given intervention is often delineated by attendance and/or involvement in a specific activity at a particular moment. However, a child's activities evolve over time and with changing contexts as they grow and encounter new life-roles. To align these shifts, both the goals and methods of interventions must adapt over time. The extent to which the intervention process is executed in a participatory manner plays an important role in achieving the ultimate and enduring objective: fostering individuals’ autonomy in selecting and adapting contexts and activities for their participation. In essence, the ultimate and enduring goal across a series of participation interventions is to empower individuals to be autonomous, capable of shaping their own participation. To realize this goal, participation in the process must be viewed as a means.

## 4 Part 2: participation as a means: enhancing involvement in participation interventions

What do we understand regarding child and family involvement at various stages of the participation intervention process, and what insights do we have into the results of their engagement in these stages? Within each of the six steps comprising the intervention process (presented in Table 1), there are opportunities to encourage the active participation of both the child and their family, facilitating the development of the skills and knowledge essential for supporting their autonomous participation. This intervention process can be viewed as an educational experience, equipping children and family with insights into how impairments and environmental adjustments may influence participation. Repeated exposure to such interventions can also nurture knowledge about impairment-environment interactions, skills in problem-solving and self-advocacy among children and care providers, which can promote future participation experiences (28).

Each step of the intervention process contains educational elements from which children and care providers can derive knowledge. In practical terms, these steps may partially overlap, occurring iteratively—for instance, transitioning between defining and explaining the problem. An integrative review on coaching in occupational therapy (31) underscores the significance of involving children and families throughout all stages of the intervention process. The review highlighted variations in the directive nature of coaching, with more directive approaches emphasizing the remediation of impairments, while less directive coaching focused on aiding clients in formulating their solutions to participation issues, often through adjustments to activities or the environment.

Coaching is further explored in a perspective paper on coaching in pediatric rehabilitation (32). By comparing three contemporary coaching approaches rooted in family-centered and capacity-building strategies, common elements emerged, including non-directive, collaborative, and reflective coaching behaviors, active listening, and a client-centered mindset. These approaches emphasized a focus on participation goals rather than body functions. While client change mechanisms and outcomes linked to capacity building and empowerment were mentioned, they were not explicitly discussed in terms of concrete strategies. Table 3 illustrates hypothetical pedagogical outcomes for the child and care providers, stemming from their involvement in various stages of the intervention process. In addition to what is displayed in Table 3, children and families also need opportunities and coaching in how to apply the knowledge and skills learned, for example, by linking impairments to environmental opportunities.

**Table 3:** Examples of hypothetical educational goals for involvement in the intervention process.

Step	i	ii	iii	iv	v	vi
Stakeholder	Identifying participation problem	Explaining participation problem	Prioritizing problem to work with and set goals	Design the method	Implement method	Evaluating outcomes
Child	Explicit knowledge about preferences	Learn how impairment and environment affect participation	Learn to reason about consequences of choices; Skills in goal setting	Learn to look for multiple explanations for participation problems	Problem solving skills and self-advocacy	Skills in self-evaluation
Family	Learn about child and family preferences	Learn about how impairment and environment affect participation	Managing family and child priorities; skills in goal setting	Learn to look for multiple explanations for participation problems	Problem solving skills and family advocacy	Skills in evaluation for solving future problems
Professional	Understand child preferences	Identify explanations for problems	Understand Family priorities; confirm outcome	Understanding of natural contexts and impacts on participation	Understand link between explanations and methods	Learn more about effective interventions

### 4.1 Therapy as a relational and participatory experience

At the core of therapeutic interactions within each stage of the intervention process lies the therapist-client relationship. In the realm of pediatric rehabilitation, a supportive relationship is characterized as a collaborative partnership that motivates and engages the client (33). Strategies for bolstering supportive relationships encompass empathetic practices such as active listening and empathic responses, as well as encouragement and guidance (34). A supportive relationship, which fosters feelings of safety and security, correlates with positive therapeutic outcomes (35). Nevertheless, feelings alone may not necessarily impart the knowledge and skills essential for the child's future participation experiences.

In their collaboration with families, Dunst et al. (6) distinguish between relational and participatory practices applied to professional-child interactions. Participatory capacity-building practices focus on how professionals utilize their supportive behaviors and routines to empower family members and children to become autonomous problem-solvers. Over time, this approach helps develop self-efficacy in terms of interacting and collaborating with professionals. Adopting relational and participatory practices are positively linked to parent self-efficacy beliefs, parental well-being, parent-child interactions, and child well-being (6).

Recognizing that relational practices remain a continuous presence throughout the entire intervention process (32), the subsequent section predominantly concentrates on the incorporation of participatory practices at every stage of the intervention process. An et al. (6) evaluated outcomes associated with utilizing participatory strategies in collaboration with families throughout the intervention process. A randomized control trial, partially built upon Dunst and Trivette’s (36) work (6), assessed the impact of employing collaborative problem-solving strategies (rooted in a participatory approach) by professionals. Parents and physical therapists, trained in a collaborative intervention process (experimental group), engaged in more interactions with each other during the planning and execution of interventions, while therapists in the comparison group focused interactions more on the children. In the experimental group, therapists displayed a higher frequency of behaviors like “seeking information,” “giving information,” and “positive behavior” (e.g., encouragement, praise, or expressions of agreement/acceptance), along with a lower frequency of “child-related behavior” (e.g., direct communication with the child or hands-on therapy) compared to therapists in the comparison group. The results demonstrated that the effect size for changes in child performance (d = 0.73) and parent satisfaction (d = 1.08) based on the Canadian Occupational Performance Measure (37) favored the experimental group. While the positive effects of implementing participatory strategies in this study were observed in terms of parents’ involvement, it is probable, though necessitating further evaluation, that the participatory principles for involvement are equally applicable in promoting children's participation within the intervention process. This approach underscores the utilization of participation in intervention to cultivate autonomy in achieving participation as an ultimate outcome.

#### 4.1.1 Identifying the participation problem

In the initial step of the intervention process (refer to Table 1), various assessments can be employed to pinpoint participation issues. These assessments can include self-rated instruments like Picture my Participation (PmP) (38), proxy ratings such as the Participation and Environment Measure for Children and Youth (PEM-CY) (39), semi-structured interviews conducted with the children themselves or their care providers, or observations. Considering the ownership of the problem is crucial, as it significantly impacts three key aspects of problem identification: (i) whether children are aware of the problem targeted by the intervention, (ii) which problem takes precedence for intervention, and (iii) whether the child's insights are valued and integrated into the intervention process. Findings from an ongoing qualitative study involving children receiving pediatric rehabilitation showed that children with disabilities often express uncertainty about why they are engaged in pediatric rehabilitation (40). These findings suggested that children's participation in the intervention process could be more extensive, and they may not be fully conscious of the problems scheduled for intervention. Increased child participation in identifying the participation issues to be addressed as well as in establishing intervention goals, enhances the likelihood of reaping the benefits of the intervention.

The significance of involving children in identifying participation problems cannot be overstated (see Table 4). In a study by Liao et al. (29), children's and parents’ identifications of three preferred activities for change were compared using ratings from an adapted version of the PmP instrument. The results revealed that in 63% of the child-parent pairs, the selections of activities for change were entirely different. Children chose activities in which they were somewhat to very involved, while parents opted for those they believed their children were less engaged in. This underscores the importance of actively involving children in the process of identifying activities they consider important for change. However, it's worth noting the shortage of self-rating tools that focus on participation (41).

**Table 4:** Important points to consider at each intervention step.

Intervention step	Important points
i. Identify the problem	The importance of involving children in identifying participation problems to change cannot be under-estimated.
ii. Generate explanations to the participation problem	• It seems more difficult for children and care providers to generate explanations to their problems than to identify problems with participation; this is an important thing for children to learn. • Professionals usually have the tools for assessing aspects of the individual, such as activity competence and self-determination, but are often not aware of instruments (or do not have the skills) for assessing the context or environment. • The explicit distinction between problem identification and problem explanation is crucial when working with participation interventions.
iii. Prioritise the problems to work with and set goals	It is important to discuss with children and care providers the consequences of prioritizing a certain problem to address. • How important is it to solve (and to whom). • How transient is the problem. • Will solving this problem have a ripple effect. • The child and the caregivers may require guidance, and an opportunity to discuss the possible consequences of prioritizing or selecting a certain participation problem to address. The discussion can center around (i) how important it is to solve the problem for the child and caregivers; (ii) how transient a certain problem is (is it specific in time, context and task); and (iii) whether solving the problem will have a ripple effect on other problems. • Goals set by children are just as valid as goals set by parents. • Constructing the GAS scale provides opportunity to investigate and discuss the expectations of children and care providers regarding the outcome of an intervention, and to track changes in expectations during the intervention period.
iv. Design the intervention	Intervention methods are usually developed based on the explanations to the problem. • When there are multiple explanations, more actions can be taken in designing the intervention method. • All explanations to a participation problem that concern impairments and activity limitations need to be supplemented by an environmental explanation. • The child and care providers are usually the experts on the activity's environment. • Coaching children and care providers about how impairments and activity restrictions are related to environmental prerequisites is important.
v. Implement the intervention	• Intervention methods that are adhered to, are probably those that are relatively easy to perform, that fit with the context in which they are applied and can be modified without losing their effectiveness. • Children and care providers themselves can modify intervention methods if they are aware of the “active ingredients” in the method. • Knowing the active ingredients is facilitated if children and care providers understand the relationship between the explanations to the problem for which a goal is set and the content of the method*.*
vi. Evaluate the outcomes	• Both intervention implementation and outcomes should be evaluated. • Outcomes can be evaluated following single, as well as a series of interventions.

How professionals engage children in utilizing the collected information about participation problems during the identification phase is equally crucial. An observational study, involving specialist nurses and physicians, examined how they used longitudinally graphed self-ratings of the quality of life when planning interventions for children with diabetes, revealing three distinct patterns of use (42). Some professionals simply read the information and commented on it, while others mentioned the name of the instrument and inquired whether the children had any comments. A few asked the children to view the data themselves and discussed the results with them before proceeding with intervention planning. Consequently, in this study, not all professionals “utilized” the information provided by the child in the intervention process to enhance participation. This represents a missed opportunity for learning and may potentially diminish the child's motivation to engage in the intervention.

#### 4.1.2 Generating explanations to the participation problem

The second step involves generating potential explanations for the identified participation problem(s). Typically, there are multiple explanations for perceived participation problems (43). Explanations can encompass various aspects, including impairments in body functions, how activities are carried out, and factors facilitating or hindering participation within the environment. For instance, when applying the fPRC framework (11), potential explanations can pertain to activity competence (such as cognition and motor skills), the individual's sense of self (including self-efficacy, self-determination, and autonomy), and their preferences (such as interests and previous experiences). Explanations may also encompass the contexts in which the person interacts with their environment, including limited resources, demands of the activity, interaction patterns and the attitudes of caregivers.

Using ICF terminology, it's important to note that most traditional assessment tools primarily focus on evaluating body functions (e.g., intelligence or gait) or activity performance (e.g., walking or talking). These assessments examine how activities are performed in comparison to typical development (e.g., language tests) or the support level required for a child to engage in an activity within a natural setting, as seen in tools like the Pediatric Evaluation of Disability Inventory (44).

These measures were initially designed to provide professionals with information about the child's body functions and limitations in carrying out activities, rather than as educational tools to assist the child and caregiver in comprehending how impairments and activity limitations influence participation. When the objective of assessment is to enhance the child and caregiver's autonomy in understanding the consequences of impairments, it's crucial to consider how the assessment process is structured and conducted, as well as how the results are explained in relation to the participation problem.

This is especially significant to prevent potential misinterpretations of assessment findings and to leverage the advantages of collaboration in the assessment process (45).

The assessment results need to be connected to the participation problem and to the environmental adjustments necessitated by the impairments. In other words, if a child identifies a participation problem related to organized leisure activities, one possible explanation might be their difficulty in comprehending information. Another explanation could be that the information presented about leisure activities was not tailored to their level of understanding. In Project TEAM by Kramer et al. (28), young individuals with disabilities were guided to formulate ideas about the necessary environmental adaptations that would enable their participation by addressing the explanations for their participation problems.

#### 4.1.3 Prioritizing the problems to work with

It is often the case that more than one participation problem is identified in the first step. However, it's not always feasible to address all these issues simultaneously due to resource constraints or the complexity of the needed interventions (17). Consequently, identified problems need to be ranked based on their importance to the child and caregivers. While this may seem straightforward, children and caregivers may require guidance and opportunity to discuss potential consequences of prioritizing or selecting a specific problem to address. Surprisingly, very few studies have specifically investigated the process of problem prioritization or how to assist children and caregivers in this process. One relevant study involves the Perceived Efficacy and Goal-setting System (PEGS), validated for use with children aged <12 years (46). PEGS guides the child through a series of steps, utilizing pictorial supports, to select a goal for skills training. In a randomized trial, Vroland-Nordstrand et al. (47) demonstrated that children can achieve goals set using PEGS. A similar approach could be used with an array of images representing activities important to children and family, followed by the PEGS methodology. Alternatively, a Talking MatsTM approach to problem prioritization might be helpful (29).

To help children choose problems to prioritize for interventions, the discussions can revolve around three key factors:

1. Importance: How crucial the problem is for children and caregivers to resolve. It's essential to consider that children and caregivers may not always share the same perspective on importance (48), so it's important to listen to the child's viewpoint.
2. Transience: Whether the problem is transient, meaning it is specific to a certain time, context, or task. Transient problems are often easy for children to grasp, but because they are limited to specific conditions, what is learned from solving them may not readily transfer to other contexts and times—a quality significant for facilitating future participation.
3. Ripple Effects: Whether solving the problem will have a cascading impact on other issues. For example, if a child seldom takes the initiative in activities, supporting the child to initiate various activities can have ripple effects on their overall participation, opening new opportunities.

These three considerations can guide children and caregivers selecting the most suitable problems for intervention.

#### 4.1.4 Setting goal/goals

A participation goal is intrinsically tied to the perceived participation problem, which can be characterized as the present state (how things are today) and the goal as the desired state (how things will be when the problem is resolved or ceases to exist). Numerous studies have explored the nature of participation-related goals and strategies for increasing the child's active involvement in goal-setting.

Studies by Klang et al. (49) and Robertson et al. (50) categorized goals set for children with complex developmental disabilities in special education and early intervention. They employed ICF-codes to represent the domains of body function, activity, and participation. In both studies, findings indicated that goals were primarily classified as activity goals focused on learning skills, with fewer goals formulated as participation goals. This raises concerns because evidence supporting the idea that activity goals, specifically instrumental goals concerning participation, automatically result in increased participation, is weak (1). In practice, it is crucial to explicitly formulate goals as participation goals, based on identified and described participation problems: that is, too much or too little attendance or involvement in particular life situations. Changing professional's practices towards participation goals is challenging because of the long-standing focus on skill development in existing rehabilitation practices as well as the lack of assessment instruments focused on identifying participation problems (22).

Several studies have explored children's active involvement in goal setting within intervention processes, and they show that children can be effectively supported to set valid goals. While these studies differ in specific instruments used to set goals, they share common finding that goals set by children are equally valid compared to those set by parents. However, it's important to consider how to support children who may face challenges in setting goals by themselves. Among the studies, only one conducted by Ullenhag et al. (4), focused on setting participation goals, while the others primarily centered on activity goals. Nevertheless, all three studies highlighted the importance of actively involving children in the goal-setting process.

Participation goals are easier understood and achieved if they are transient, meaning they are specific in terms of time, context, and task. To set goals, professionals should collaborate with children and caregivers to clearly define what goal attainment would look like. Goal attainment should be described in everyday language, illustrating the difference between the current state and the desired state. For example, if the current situation is that the child plays with classmates at recess less than once per school day, the goal could be set as playing with classmates at least twice out of four breaks every school day.

One effective tool for setting concrete goals is the Goal Attainment Scale (GAS) (51), which has been widely used in pediatric research. GAS scales describe the gap between the current situation and the desired goal and specify the time frame for evaluating goal attainment. Maintaining motivation is challenging when it takes a long time to perceive a change in the goal or to reach it. Therefore, for younger children and children with cognitive impairments, it's crucial to primarily set short-term goals, ideally within one to two months. GAS can be developed in collaboration with children and caregivers, and the process of constructing the scale provides an opportunity to explore and discuss their expectations regarding the intervention's outcome. Depending on the goal and intervention method, the time frame for goal attainment and the specific steps within a GAS scale can be determined. GAS can also be used for longer time periods to evaluate ultimate goals, such as the use of skills in managing one's own participation.

#### 4.1.5 Designing the intervention method and plan

Developing effective intervention methods requires deep understanding of the explanations for the identified problem. When multiple explanations for a participation problem exist, it becomes easier to generate effective intervention methods. Analyzing how different explanations are related to each other is crucial in this process.

In addition to understanding the explanations related to impairments and activity restrictions, it's equally important to consider environmental explanations. The environment plays a significant role in shaping participation experiences.

For example, consider the participation problem: “I (Tim) seldom play with peers in outdoor activities in preschool.” This problem can be explained in multiple ways. Tim may have a motor impairment affecting his ability to walk and climb on certain surfaces. Another explanation could be that Tim lacks mobility aid adapted for outdoor play. A third explanation may be that the preschool staff don't actively create outdoor activities that accommodate Tim's mobility challenges.

Multiple explanations provide more opportunities for designing effective intervention methods. The child and care providers have unique insights into the environmental characteristics of activity settings, making it essential to coach them on how impairments and activity restrictions relate to the environmental prerequisites. This collaborative approach can lead to more comprehensive and successful interventions.

#### 4.1.6 Implementing the intervention

Implementing interventions for children in everyday activity settings is essential for achieving participation goals, although posing unique challenges. In such everyday settings, professionals are not always present to provide direct guidance. Therefore, strategies for offering feedback and coaching at a distance are crucial. However, research on these distance coaching strategies in pediatric rehabilitation and school settings is limited (9).

There is growing interest in coaching as a therapeutic approach for supporting intervention processes, including helping children and families work with intervention methods after setting goals. While coaching has been explored, there is still limited knowledge on how to effectively support children and families in implementing intervention methods in their everyday activities.

Some examples exist, such as an internet-based support and coaching model for adolescents with attention deficit hyperactivity disorder (ADHD) and autism, where coaching sessions were conducted via internet chat functions. This model resulted in significant improvements in behavior regulation outcomes (52). Another study investigated the impact of providing feedback to children and adolescents with autism based on self-report outcome measures, leading to more substantial goal attainment in the intervention (53).

These studies suggest that distance coaching and feedback can be effective in supporting children and families in implementing intervention methods in real-life situations. Further research in this area is needed to develop and refine strategies for effective distance coaching and feedback in pediatric rehabilitation and school settings.

Intervention methods that are easily adhered to tend to be those aligning well with their applied context and allowing for modifications without compromising their efficacy. Our clinical experience underscores that children and caregivers typically gauge a method's utility in their daily routines within a few days of implementation. However, scheduling a professional follow-up at one to two months’ intervals poses a risk of children and caregivers disengaging from the intervention. A more frequent check-up schedule, perhaps once or twice a week using remote methods, could offer a solution. Children and caregivers can adapt intervention methods themselves, especially if they grasp the “active ingredients” within a given method. Understanding these active components becomes easier when children and caregivers comprehend the link between the problem explanations behind their goal-setting and the method's content. This insight enables them to consider modifications that preserve the active ingredients. Graham et al. (54) documented three case studies illustrating coaching strategies that assist parents in achieving better alignment among child characteristics, environmental conditions, and task requirements by asking questions that promote parental comprehension of the activity.

Intervention methods that influence both children with impairments and individuals in their social environments tend to yield more sustainable outcomes. These transactional effects necessitate changes in both the child and the people within the environment over time as a result of the intervention. Hsieh et al. (23, 24) have detailed the transactional effects observed when introducing eye gaze technology to children with severe multiple impairments through a series of single subject designs aimed at enhancing participation. After the introduction of eye gaze devices for communication, children displayed greater initiative in learning activities and play. Simultaneously, interaction patterns of their adult communication partners underwent a transformation, transitioning from a high number of communication initiatives to a lower count, accompanied by a higher proportion of responses. These shifts, in turn, prompted the children to increase their communication initiation frequency even further.

#### 4.1.7 Evaluating implementation and outcomes/effects

The evaluation of interventions should encompass both implementation and outcomes. For individual interventions, adherence to the prescribed method can be gauged using visual analogue scales, with adherence rated on a scale ranging from 0 (not at all) to 100 (as planned/always). Regularly assessing the feasibility of method implementation is also crucial (52). Assessing adherence to a series of interventions is intricate and may necessitate multiple instruments, as it involves not only measuring adherence to individual interventions but also appraising the overall pattern of all interventions (17).

Assessments of goal attainment (outcomes) is applicable to both individual interventions and sequences of interventions. In the case of single interventions, the objectives typically target transient outcomes, such as increased engagement in specific activities, and can be assessed using a Goal Attainment Scale (GAS) that measures aspects of participation like attendance and involvement. However, for a sequence of interventions, desired outcomes (goals) should be aligned with enduring or ultimate results for the child, such as acquiring the necessary skills to independently shape their participation across various life domains. Evaluating outcomes of a series of interventions may involve employing participation measures, which focus on changes in participation profiles and levels across various activities [e.g., (55)]. Alternatively, assessments may emphasize person-level factors crucial for overall participation, like self-determination (56) or problem-solving skills for identifying environmental elements affecting participation (28). Nevertheless, detecting these changes and attributing them to a sequence of interventions is challenging because constructs like self-determination and problem-solving skills encompass latent aspects that incorporate more concrete concepts such as goal-setting skills or the ability to analyze environmental characteristics. Consequently, children must engage in the development of these skills over extended periods before reliable changes in self-determination and problem-solving skills can be observed (57). As transformations unfold over prolonged timeframes, distinguishing whether they result from a sequence of interventions or other factors can be challenging (57).

## 5 What do we not know?

Research is needed now to:

i. Investigate impact of prolonged intervention implementation on children's autonomy in shaping their participation in life.
ii. Assess sustainable and transactional changes in the environment due to children's increased participation.
iii. Explore societal and community-level changes required to support enhanced participation for children with impairments. Include individuals with disabilities at all levels for meaningful outcomes.

It is essential to involve people with disability at all ecological levels from the individual to society to achieve ultimate and enduring participation outcomes.

## 6 Summary

Connections between participation, intervention, and outcomes must be clear in the treatment process. Participation is a comprehensive concept, serving as both an outcome and a method. Using participation as a method within the intervention process is essential for achieving positive enduring participation outcomes (i.e., increased participation in several activities also outside those explicitly addressed in planned interventions). To establish intermediate and ultimate participation outcomes, a series of interventions is necessary. These interventions should define the desired results of involving children and caregivers in each step of the intervention process in every intervention, from identifying participation issues to assessing the implementation and goal achievement of participation interventions. Structuring roles and responsibilities of children, caregivers, and professionals in the intervention process is a crucial intervention to foster intermediate and ultimate participation outcomes. To attain lasting outcomes encompassing both children's and caregivers’ skills and environmental adaptations, we must focus on ultimate goals.

## Data Availability

The original contributions presented in the study are included in the article, further inquiries can be directed to the corresponding authors.
